# The association between cumulative distal and proximal adversity with depression and anxiety among township grassroots civil servants in China

**DOI:** 10.3389/fpsyt.2025.1480559

**Published:** 2025-11-06

**Authors:** Xiong Zhu, Yawen Zheng, Mengting Wang, Wenqian Jian, Hong Pan, Li Chen, Xiaoyue Liu

**Affiliations:** 1 Department of Psychiatry, The Third People’s Hospital of Huzhou Municipal, The Affiliated Hospital of Huzhou University, Huzhou, China; 2 Lishui Second Affiliated Hospital of Wenzhou Medical University, Lishui, China; 3 School of Mental Health, Wenzhou Medical University, Wenzhou, China; 4 The Affiliated Wenzhou Kangning Hospital, Wenzhou Medical University, Wenzhou, China; 5 School of Medical Humanities and Management, Wenzhou Medical University, Wenzhou, China

**Keywords:** depression, anxiety, township grassroots civil servants, distal and proximal adversity, cumulative, China

## Abstract

**Background:**

Depression and anxiety are significant public health concerns among township grassroots civil servants, the largest segment of China’s civil service. This study aims to investigate the prevalence of depression and anxiety within this population, identify key distal and proximal adversity factors using the Developmental Adaptation Model, explore the association between cumulative adversity and mental health outcomes, and analyze the underlying pathways of association.

**Methods:**

A cross-sectional study of 1,275 township grassroots civil servants collected data on demographics, distal adversities (e.g., left-behind experiences, emotional abuse), proximal adversities (e.g., parent-child conflict, divorce intentions, work stress), depression and anxiety using self-report questionnaires. Logistic regression was used to identify predictors, while ANOVA and hierarchical regression were used to examine the functional form of the relationship between cumulative adversity and mental health. Finally, mediation analysis was conducted to explore the role of proximal adversity in linking distal adversity to mental health outcomes.

**Results:**

The prevalence of depression and anxiety among township grassroots civil servants was high, at 36.7% and 29.6%, respectively. Multiple distal and proximal factors were significantly associated with both outcomes. Among distal adversities, domestic violence (aOR = 3.42) and emotional abuse (aOR = 2.89) were the strongest correlates of depression; among proximal adversities, work stress (aOR = 5.02) and economic poverty (aOR = 4.92) had the most substantial associations. Cumulative adversity was significantly and positively associated with both depression and anxiety (*p* < 0.001), showing a clear linear pattern. Mediation analysis revealed that the effect of cumulative distal adversity on both depression and anxiety was fully mediated by cumulative proximal adversity.

**Conclusion:**

This study highlights an alarming prevalence of depression and anxiety among township grassroots civil servants. The findings underscore that while early life adversity creates a foundation of risk, current (proximal) stressors are the primary mechanism through which this risk translates into psychological distress. These insights can help government agencies develop more effective, targeted interventions by focusing on mitigating current work and life pressures.

## 1 Introduction

Depression and anxiety represent major global public health challenges and are leading causes of disability worldwide (1, 2). Within China, township grassroots civil servants constitute a particularly vulnerable yet often overlooked population. As the largest segment of China’s civil service, they serve as the crucial “last mile” link between national policies and hundreds of millions of citizens. Their mental well-being is thus directly tied to the success of grassroots governance and broader social stability (3–5). However, this unique position exposes them to multiple and intense occupational stressors. Compared to many other professions, they face substantial workloads, intense competition, and challenging interpersonal dynamics, while simultaneously managing complex and often conflicting public demands with limited resources. This often places them in a “sandwich” position of high demands and low control (6, 7), creating a high-risk environment for developing mental health disorders.

The severity of this issue is confirmed by a growing body of evidence. By comparison, among medical professionals, 22.0% reported depressive symptoms and 17.9% anxiety (8); nationwide, 17.7% of teachers showed signs of anxiety (9). In contrast, China’s National Mental Health Development Report (2017–2018) indicated that among Chinese civil servants, the proportions experiencing moderate to high levels of anxiety and depression reached 35.2% and 33.2%, respectively (CNMEHDR, 2019). Specifically, a study targeting grassroots civil servants estimated the prevalence of depressive and anxiety symptoms to be as high as 37.25% and 38.06%, respectively (10), highlighting the severe mental health challenges faced by this group. Given cultural factors and occupational stigma, under-reporting is common; thus, the true mental health burden is likely even higher. Therefore, a thorough investigation into the risk factors underlying this severe situation is essential for developing effective prevention and intervention strategies.

Previous research on the mental health of township grassroots civil servants has predominantly focused on proximal adversities, such as immediate work pressure and family issues (6, 11–14). However, this narrow focus overlooks the foundational role of distal adversities-adverse experiences in childhood that can create long-term psychological and biological vulnerabilities. Based on the Cognitive Vulnerability-Stress Theory and Emotion Regulation Theory, early traumas can impair emotion regulation and foster negative cognitive patterns, increasing susceptibility to adult stress (15). Neurobiological evidence shows that early life stress is known to program long-term dysregulation of the hypothalamic-pituitary-adrenal (HPA) axis and alter brain structures critical for mood regulation (16). Current proximal stressors then act on this pre-existing vulnerability, perpetuating dysregulation and contributing to the neurochemical imbalances that underpin depression and anxiety (17). Despite the established importance of factors from both life stages, a significant research gap remains. Few studies have systematically integrated both distal and proximal adversities within a coherent, life-course framework to understand the mental health of this specific, high-stress population. This oversight limits the development of truly comprehensive prevention strategies. Therefore, the present study aims to address this gap by examining the combined and cumulative impact of both distal and proximal adversities on depression and anxiety among township grassroots civil servants.

The Developmental Adaptation Model and Life Course Theory can be applied to understand mental health among township grassroots civil servant by emphasizing the combined impact of distal and proximal adversity on depression and anxiety outcomes. The former posits that mental health is shaped by the interplay of risk factors across different life stages (distal and proximal) (18), while the latter emphasizes that early life experiences have long-term and profound effects on developmental trajectories in adulthood (19). Based on this foundation, we selected specific adversity factors to assess their impact on the mental health of township grassroots civil servants.

Regarding distal adversity factors, we focus on core childhood experiences that have been proven to shape long-term psychological vulnerability (20–22). In China, the left-behind experience is a unique social phenomenon arising from large-scale rural-to-urban migration. Many grassroots civil servants come from rural backgrounds where parental absence and associated emotional neglect are common, which has been shown to increase the risk of mental health in adulthood (23, 24). Severe childhood traumas like emotional and physical abuse can damage an individual’s self-esteem and emotion regulation skills, weakening their resilience to occupational stress in adulthood (25). Unstable family environments, such as witnessing domestic violence and parental divorce, disrupt a child’s sense of security, heightening their sensitivity to adult interpersonal conflicts and pressures (26). Furthermore, childhood poverty is not merely material deprivation but also a chronic stressor that can influence emotion regulation abilities, with negative consequences persisting into adulthood (27). These distal factors are critically important as they reflect universal developmental principles that apply to civil servants just as they do to the general population.

Regarding proximal adversity factors, our selection targets the specific occupational and life stressors of the civil servant population. Work stress is central, as these officials face heavy workloads and high accountability, aligning with “high-demand, low-control” and “effort-reward imbalance” models that strongly predict poor mental health (10, 28). This stress often spills over into family life, manifesting as parent-child conflict and divorce intention (29). Despite job stability, grassroots civil servants often experience economic pressure and a sense of relative deprivation due to modest salaries (30). Moreover, smoking and drinking were included as they represent common, yet maladaptive, coping strategies reinforced by the networking culture prevalent in grassroots administration (31). Finally, as frontline policy implementers, grassroots civil servants frequently face public misunderstanding and even negative judgment; this perceived discrimination constitutes a unique psychosocial stressor that can undermine their professional identity (32).

Beyond simply identifying these risk factors, it is crucial to understand their combined role. The concept of cumulative risk has fundamentally shifted the paradigm of psychopathology research, moving the focus from single risk factors to the aggregation of adversities (33). This model posits that the sheer number of risk factors an individual is exposed to is a more potent predictor of negative outcomes than the nature of any specific risk (34, 35). Extensive research has demonstrated a strong, dose-response relationship between the accumulation of life stressors and the prevalence and severity of depression and anxiety in adulthood (36). However, the precise nature of this relationship is a subject of ongoing investigation. Numerous studies have suggested that the relationship between cumulative risk and individual development may take three different functional forms (37–39). The positive acceleration model posits that the impact of each risk factor intensifies as other risks accumulate. Conversely, the negative acceleration model proposes that the influence of each additional risk factor lessens. Finally, the linear model assumes a direct proportional relationship, where each additional risk factor contributes an equal amount of risk, demonstrating a “gradient effect”. Therefore, analyzing the functional form is a crucial step in clarifying how adversity impacts mental health.

Furthermore, it is essential to explore the mechanisms linking these factors. Research has shown that distal adversity can have a significant relationship with mental health, both directly and indirectly (40). Distal adversity can create or worsen conditions in an individual’s immediate environment, such as increasing financial strain or disrupting social networks, which can then be associated with proximal adversity. In turn, proximal adversity can have a direct association with depression and anxiety, as individuals may struggle to cope with the immediate stressors in their lives (41). Therefore, it is important to understand the mediating role of proximal adversity in the relationship between distal adversity and depression and anxiety. By identifying the specific pathways through which distal adversity affects depression and anxiety, we can develop more targeted and effective interventions to support township grassroots civil servant in the face of adversity.

**Figure 1:**
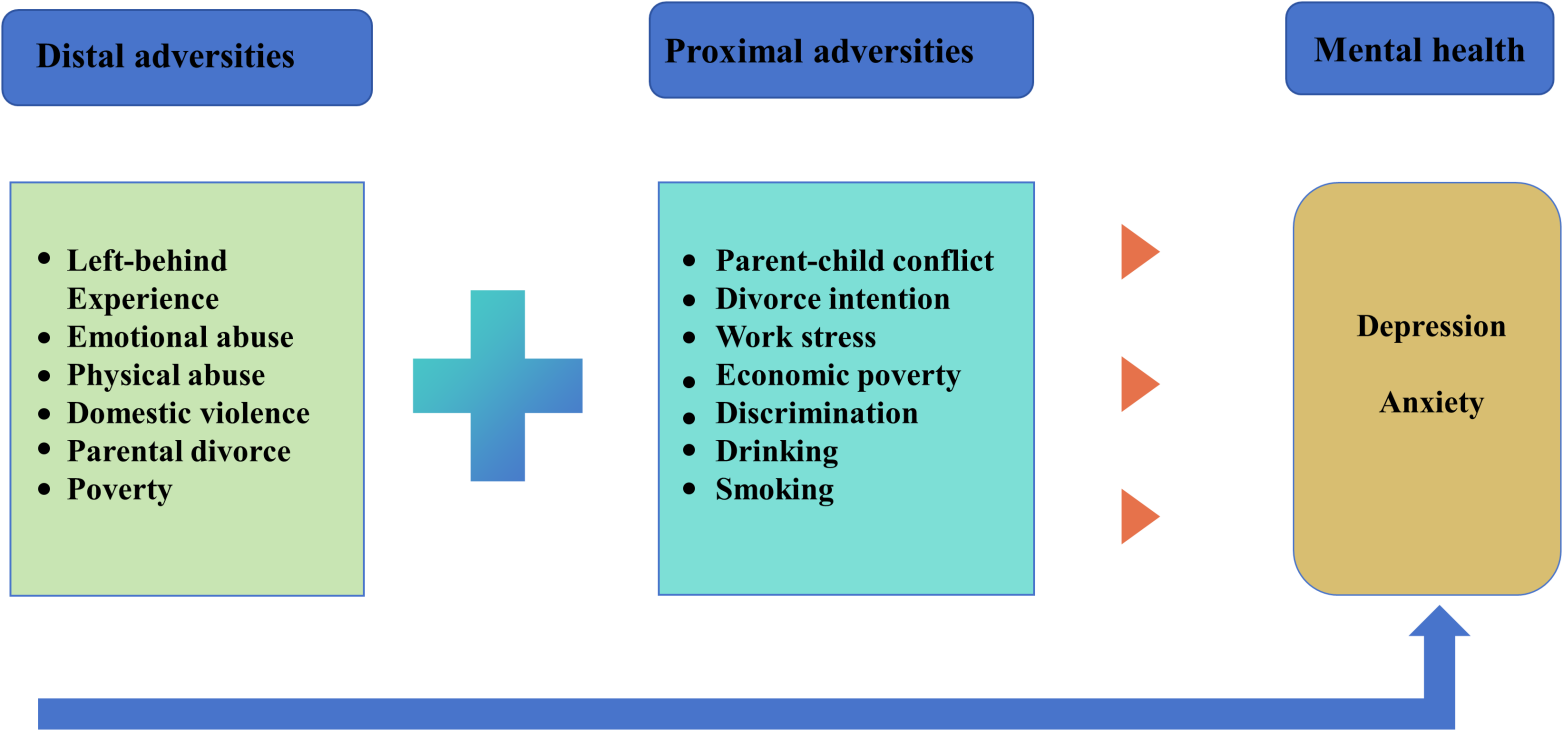
The distal and proximal adversity of depression and anxiety.

Therefore, this study aims to (1): investigate the current prevalence of depression and anxiety among township grassroots civil servants in China (2); identify key distal and proximal risk factors (3); determine the functional form of their cumulative association on mental health; and (4) explore the mediating mechanism through which distal adversity is associated with mental health via proximal adversity. Based on this framework, we formulated the following hypotheses (**Figure 1**):

Hypothesis 1 (H1): Both distal and proximal adversities will be significantly and positively associated with the prevalence of depression and anxiety symptoms.

Hypothesis 2 (H2): A positive linear relationship will exist between cumulative adversity and the severity of symptoms, demonstrating a “gradient effect.

Hypothesis 3 (H3): Cumulative proximal adversity will fully mediate the relationship between cumulative distal adversity and mental health outcomes.

## 2 Method

### 2.1 Data and sampling

This cross-sectional study was conducted from March to April 2022. The target population was township grassroots civil servants from the middle and lower reaches of the Yangtze River in China. This region was selected because it is one of the country’s most economically developed areas, with a high concentration of this population. Furthermore, its recent significant social and economic changes may have an impact on the mental health of civil servants.

A multi-stage stratified random sampling method was used to select the study sites. First, the region was stratified into three levels based on economic development (high, medium, and low). Two provinces were randomly selected from each stratum (totaling six provinces). Next, three counties were randomly selected from each province (totaling 18 counties), and two townships were randomly selected from each county, resulting in a final sample of 36 townships. The inclusion criteria for participants were as follows (1): currently employed as a full-time, township-level civil servant; (2) aged 18 years or older; and (3) provided informed consent to participate. Exclusion criteria included: civil servants who were on probation or in a trainee period.

The recruitment process was conducted as follows. Our research team established contact with the organizational departments of various government units through a government-supported mental health care program. Upon receiving their full support and official permission, designated liaisons within each township government were asked to assist in distributing the link to the online questionnaire. These liaisons were instructed to emphasize to potential participants that their involvement was completely voluntary and that all responses would remain anonymous and confidential. The online questionnaire platform first presented a detailed informed consent form, outlining the study’s objectives, procedures, potential risks and benefits, and the right to withdraw at any time. Only after providing electronic consent could the participants proceed to the questionnaire.

Finally, we randomly selected 38 township grassroots civil servants from each of the selected townships, resulting in a total sample size of 1,368 participants. Of these, 1,275 questionnaires were valid, resulting in an effective rate of 88.54%. The questionnaires were anonymous and all participants took part in the study voluntarily. This study was conducted in compliance with the Helsinki Declaration and was reviewed and approved by the Ethics Committee of Wenzhou Medical University (approval number: 2023-023). Written informed consent was obtained from each participant before starting any investigation related to the study.

### 2.2 Measurements

#### 2.2.1 Socio-demographic factors

The socio-demographic factors include gender, age, educational level and location of residence (rural or urban). Educational level is categorized as college below and college or above.

#### 2.2.2 Distal adversity factors

This research utilized the revised Adverse Childhood Experiences Questionnaire (ACEQ-R) to evaluate distal adversity (42). The questionnaire assesses fourteen aspects of ACEs: parental emotional abuse, parental physical abuse, sexual assault, parental emotional neglect, parental physical neglect, witnessing domestic violence, family member drug/alcohol problems, family member mental illness, parental separation/divorce, family member incarceration, peer victimization, peer isolation/rejection, exposure to community violence, and low socioeconomic status. For example “Was there a period of 2 or more years when your family was very poor or on public assistance?”. It was validated in a Chinese sample by Cao et al. (2018), who reported a Cronbach’s alpha coefficient of 0.83 for the scale (43). This test has been widely used for measuring childhood adversity in Chinese individuals in previous studies (44, 45). In this study, the Cronbach’s alpha for the scale was 0.73. We selected five of these variables (emotional abuse, physical abuse, domestic violence, parental divorce and poverty), and added whether they had experienced being left-behind to form the distal adversity factors. Responses to each item were either “yes” or “no.” Responding “yes” will be coded as “1” and be considered to have corresponding ACEs; responding “no” will be coded as “0”.

#### 2.2.3 Proximal adversity factors

Proximal adversity factors include parent-child conflict, divorce intention, work stress, economic poverty, drinking, smoking and perception of discrimination. Parent-child conflict: By question “My child and I seem to be in constant conflict”. Item is scored on a 5-point Likert scale, ranging from 1 (“extremely not conform”) to 5 (“extremely conform”). Divorce intention: Asked if there was any intention to divorce, on a scale of 1 to 4, never to recently. Work stress: This question “I feel stressed going to work”. Item is scored on a 7-point Likert scale, ranging from 1 (“never”) to 7 (“every day”). Economic poverty: Answer yes or no to the question “Do you feel poor”. Responding “yes” will be coded as “1” and be considered to have poverty; responding “no” will be coded as “0”. Drinking and smoking: The question asked were, “Have you ever drunk alcohol?” and “Have you ever smoked?”. In the current study, the participants were asked to indicate how often they experienced each symptom in the period of one year on a 5-point Likert scale (1 = never, 2 = rarely, 3 = sometimes, 4 = often and 5 = very often). Perception of discrimination: This question is “most people look down on the township grassroots civil servants”. A 4-point scale is used, from strongly agree to strongly disagree.

#### 2.2.4 Depression and anxiety

Depression and anxiety was measured by DASS-21 with 7 item for each subscale and participants responded to it by indicating how much a statement applied to them over the past week on a 4-point Likert scale (46). For example “I was unable to become enthusiastic about anything”. Each scale with higher scores indicating more severe symptom. Following the official scoring guidelines established in the DASS manual (46) and clinical research (47), depression scores were categorized into normal (0–9), mild depression (10-13), moderate depression (14-20) and severe depression (21 and above). Anxiety scores were categorized into normal (0-7), mild anxiety (8-9), moderate anxiety (10-14) and severe anxiety (15 and above). It was validated in a Chinese sample by Chan et al. (2016), who reported a Cronbach’s alpha coefficient of 0.83 and 0.82 for depression and anxiety, respectively (48). It has been extensively applied in Chinese samples (48, 49) and in our study the Cronbach a coefficients for depression and anxiety were 0.92 and 0.93, respectively.

### 2.3 Statistic analysis

All statistical analyses were performed using IBM SPSS Statistics package (version 25.0), with all P-values being two-tailed and a significance level set at α = 0.05. The analytical process was structured into several sequential stages: (1) data cleaning and pre-processing, (2) descriptive and bivariate analyses, (3) logistic regression analyses, (4) cumulative adversity analyses, and (5) mediation analyses.

#### 2.3.1 Data cleaning and pre-processing

First, prior to the main analyses, the data were screened for outliers and missing values. The rate of missing data was minimal (< 5% for any variable), and cases with missing data were excluded from analyses using listwise deletion. No extreme outliers that would unduly influence the results were identified.

The DASS-21 scores were used both for descriptive classification and as a binary outcome for logistic regression. For descriptive purposes, scores were categorized into normal, mild, moderate, and severe. For the logistic regression analysis, a binary variable was created: participants with scores in the mild, moderate, or severe ranges (a depression score ≥ 10; an anxiety score ≥ 8) were classified into the “symptomatic group” (coded as 1), while those in the normal range were classified into the “non-symptomatic group” (coded as 0). This approach allows for the identification of risk factors associated with the presence of any level of clinical symptomatology (50–52).

For proximal adversity factors measured on scales without established clinical cutoffs (e.g., work stress), we dichotomized the variables for logistic regression analysis. Following a common methodology for risk factor analysis known as extreme group analysis, we used percentile-based cutoffs to create distinct groups for comparison. Participants scoring in the top quartile (≥ 75th percentile) on a given risk factor were classified as “at risk” (coded as 1), while the remaining participants were classified as “not at risk” (coded as 0). This method is effective for identifying the impact of high exposure to specific stressors (53, 54).

#### 2.3.2 Descriptive and bivariate analyses

Descriptive statistics were used to summarize the demographic characteristics of the participants, as well as the prevalence of depression, anxiety, and adversity factors. Continuous variables were presented as means and standard deviations, while categorical variables were presented as frequencies and percentages. For associations between categorical variables, we used the chi-square (χ²) test.

#### 2.3.3 Logistic regression analyses

First, multicollinearity was assessed using the variance inflation factor (VIF), and all values were found to be below the threshold of 5, suggesting it was not a concern. Then, we entered the demographic variables that were significantly associated with the outcomes in the bivariate analyses as covariates. These included age, gender, marital status, and education level. In the second step, we added the distal and proximal adversity factors. This approach allowed us to assess the unique contribution of adversity factors after controlling for demographic confounders. The results are presented as odds ratios (ORs) with their 95% confidence intervals.

#### 2.3.4 Cumulative adversity analyses

The scores of all risk factors were subsequently summed to calculate the cumulative distal and proximal risk index (55, 56). The variance analysis was used to examine the outcomes of depression and anxiety among township grassroots civil servants with different levels of adversity. We then performed a hierarchical linear regression analysis. Multicollinearity was assessed using the variance inflation factor (VIF), and all values were found to be below the threshold of 5, suggesting it was not a concern. The analysis included depression and anxiety as the dependent variable, gender, age, education and household registration as control variables and cumulative risk (linear term) as the predictor variable. A quadratic term was added to the linear term to explore the relationship further. If the regression coefficient of the quadratic term is not significant, it signifies a linear pattern. Conversely, if the regression coefficient is significant, it indicates a nonlinear relationship between cumulative risk and the outcome variable.

#### 2.3.5 Mediation analyses

Before mediation analyses, we checked for multicollinearity using the variance inflation factor (VIF). All VIF values were below 5, indicating that multicollinearity was not a significant concern. We perform the Harman’s single-factor test (57) and the results showed that there are 6 factors with eigenvalues greater than 1, the first factor explained 37.06% of the variation, which was lower than the critical value of 40%. Therefore, the common method deviation had little effect on the following analyses. Then, we used the Pearson correlation to analyze the relationship among cumulative distal adversity, cumulative proximal adversity, depression and anxiety.

After controlling gender, age, education and household registration, Model 4 of SPSS Process 3.5 was used (Hayes, 2013) to test the mediation model with cumulative distal adversity as independent variable, cumulative proximal adversity as mediating variable, and depression and anxiety as dependent variables. To robustly test the significance of the indirect effect, we employed the bootstrapping method. A total of 5,000 bootstrap samples were used to generate 95% bias-corrected confidence intervals (CIs).

## 3 Results

### 3.1 Socio-demographic characteristics of participants

The final sample consisted of 1,275 participants, with a near-equal gender distribution (50.30% female). The age breakdown reveals that 539 (42.3%) of participants fell in the 31–40 age group, 391 (30.70%) were below 31 years old, and 345 (27.10%) were over 40 years old. Regarding education, 581 (45.60%) of participants had an education level of college and above, while 694 (54.40%) had an education level of college below. Furthermore, 1071 (84%) of respondents lived in rural areas, while 204 (16%) resided in urban areas.

### 3.2 Prevalence and pattern of depression and anxiety

Based on depression severity, the overall prevalence of depression among township grassroots civil servants was 36.7%. Specifically, 807 individuals (63.3%) were classified as normal, 159 (12.5%) as mild, 207 (16.2%) as moderate, and 102 (8.0%) as severe cases of depression. Regarding anxiety, the overall prevalence was 37.8%, with 897 individuals (70.4%) categorized as normal, and 51 (4.0%), 188 (14.7%), and 139 (10.9%) experiencing mild, moderate, and severe anxiety, respectively.

### 3.3 The factors associated with depression and anxiety

Logistic regression analysis revealed that both distal and proximal adversities were significantly associated with depression and anxiety (see **Tables 1**, **2**). Among distal factors, domestic violence, emotional abuse, and parental divorce showed the strongest associations with depression. Among proximal factors, work stress and economic poverty emerged as the most potent predictors for depression. For anxiety, parent-child conflict and work stress were the most significant predictors. Additionally, substance use and perceived discrimination were significantly associated with both outcomes. 

**Table 1:** Characteristics of depression of the participants (N = 1275).

Variable	Depression		χ2	*P*	aOR (95%CI)	*P*
	No (n = 807) n (%)	Yes (n = 468) n (%)				
Distal adversities						
Left-behind experience			5.26	0.024		
Yes	145 (18.0)	109 (23.3)			1.00 (reference)	
No	662 (82.0)	359 (76.7)			1.31 (0.98, 1.74)	0.060
Emotional abuse			47.91	< 0.001		
No	727 (90.1)	354 (75.6)			1.00 (reference)	
Yes	80 (9.9)	114 (24.4)			2.89 (2.11, 3.96)	< 0.001
Physical abuse			12.73	< 0.001		
No	763 (94.5)	417 (89.1)			1.00 (reference)	
Yes	44 (5.5)	51 (10.9)			2.05 (1.34, 3.13)	0.001
Domestic violence			16.69	< 0.001		
No	791 (98.0)	438 (93.6)			1.00 (reference)	
Yes	16 (2.0)	30 (6.4)			3.42 (1.83, 6.36)	< 0.001
Parental divorce			15.88	< 0.001		
No	773 (95.8)	422 (90.2)			1.00 (reference)	
Yes	34 (4.2)	46 (9.8)			2.43 (1.53, 3.87)	< 0.001
Poverty			6.18	0.015		
No	642 (79.6)	344 (73.5)			1.00 (reference)	
Yes	165 (20.4)	124 (26.5)			1.42 (1.08, 1.86)	0.010
Proximal adversities						
Parent-child conflict			96.61	< 0.001		
No	696 (86.2)	292 (62.4)			1.00 (reference)	
Yes	111 (13.8)	176 (37.6)			4.02 (3.03, 5.32)	< 0.001
Divorce intention			155.85	< 0.001		
No	683 (84.6)	245 (52.4)			1.00 (reference)	
Yes	124 (15.4)	223 (47.6)			3.42 (2.57, 4.55)	< 0.001
Work stress			178.47	< 0.001		
No	614 (76.1)	180 (38.5)			1.00 (reference)	
Yes	193 (23.9)	288 (61.5)			5.02 (3.91, 6.44)	< 0.001
Economic poverty						
No	676 (83.8)	237 (50.6)	159.87	< 0.001	1.00 (reference)	
Yes	131 (16.2)	231 (49.4)			4.92 (3.77, 6.42)	< 0.001
Drinking			76.75	< 0.001		
No	707 (87.6)	315 (67.3)			1.00 (reference)	
Yes	100 (12.4)	153 (32.7)			3.55 (2.63, 4.81)	< 0.001
Smoking			24.38	< 0.001		
No	624 (77.3)	302 (64.5)			1.00 (reference)	
Yes	183 (22.7)	166 (35.5)			2.15 (1.61, 2.87)	< 0.001
Perception of discrimination			22.42	< 0.001		
No	319 (39.5)	249 (53.2)			1.00 (reference)	
Yes	488 (60.5)	219 (46.8)			1.70 (1.34, 2.15)	< 0.001

**Table 2:** Characteristics of anxiety of the participants (N = 1275).

Variable	Anxiety		χ2	*P*	aOR (95%CI)	*P*
	No (n = 897) n (%)	Yes (n = 378) n (%)				
Distal adversities						
Left-behind experience			1.78	0.192		
Yes	170 (19.0)	84 (22.2)			1.00 (reference)	
No	727 (81.0)	294 (77.8)			0.85 (0.63, 1.15)	0.319
Emotional abuse			28.89	< 0.001		
No	792 (88.3)	289 (76.5)			1.00 (reference)	
Yes	105 (11.7)	89 (23.5)			2.31 (1.70, 3.19)	< 0.001
Physical abuse			13.67	< 0.001		
No	846 (7.1)	334 (88.4)			1.00 (reference)	
Yes	51 (5.7)	44 (11.6)			2.15 (1.40, 3.29)	< 0.001
Domestic violence			13.96	< 0.001		
No	876 (97.7)	353 (93.4)			1.00 (reference)	
Yes	21 (2.3)	25 (6.6)			2.97 (1.63, 5.39)	< 0.001
Parental divorce			16.95	< 0.001		
No	857 (95.5)	338 (89.4)			1.00 (reference)	
Yes	40 (4.5)	40 (10.6)			2.45 (1.54, 3.89)	< 0.001
Poverty			11.66	0.001		
No	717 (79.9)	269 (71.2)			1.00 (reference)	
Yes	180 (20.1)	109 (28.8)			1.66 (1.25, 2.19)	< 0.001
Proximal adversities						
Parent-child conflict			111.48	< 0.001		
No	767 (85.5)	221 (58.5)			1.00 (reference)	
Yes	130 (14.5)	157 (41.5)			4.53 (3.41, 6.02)	< 0.001
Divorce intention			131.16	< 0.001		
No	736 (82.1)	192 (50.8)			1.00 (reference)	
Yes	161 (17.9)	186 (49.2)			4.92 (3.74 - 6.47)	< 0.001
Work stress			122.25	< 0.001		
No	646 (72.0)	148 (39.2)			1.00 (reference)	
Yes	251 (28.0)	230 (60.8)			3.928 (3.02, 5.10)	< 0.001
Economic poverty						
No	718 (80.0)	195 (51.6)	105.92	< 0.001	1.00 (reference)	
Yes	179 (20.0)	183 (48.4)			3.82 (2.93, 4.9)	< 0.001
Drinking			147.89	< 0.001		
No	733 (81.7)	182 (48.1)			1.00 (reference)	
Yes	164 (18.3)	196 (51.9)			2.508 (1.854 - 3.392)	< 0.001
Smoking			21.26	< 0.001		
No	685 (76.4)	241 (63.8)			1.00 (reference)	
Yes	212 (23.6)	137 (36.2)			2.21 (1.67, 2.99)	< 0.001
Perception of discrimination			9.12	0.002		
No	375 (41.8)	193 (51.1)			1.00 (reference)	
Yes	522 (58.2)	185 (48.9)			1.44 (1.13, 1.84)	0.003

### 3.4 The cumulative adversity on depression and anxiety

**Figure 2:**
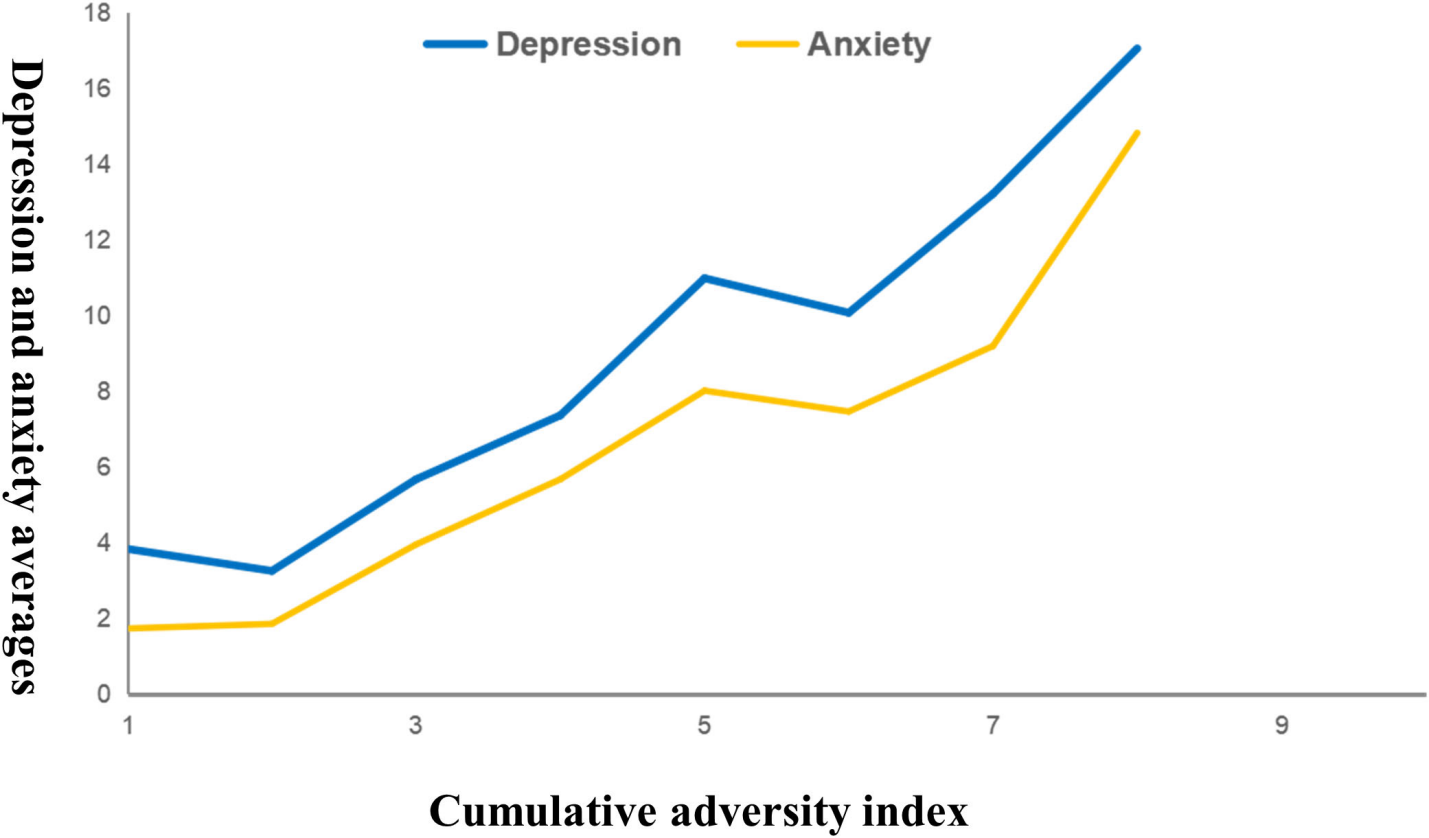
The relationship models between the number of adversities at depression and anxiety.

As detailed in **Table 3**, participants who experienced a greater number of adversities reported significantly higher levels of depression and anxiety. Further regression analysis confirmed that the cumulative risk had a significant, positive linear relationship with both depression and anxiety, even after controlling for demographic factors (*p* < 0.001). The non-significant quadratic term indicates that this relationship is best represented as a steady, additive increase in risk (see **Table 4** and **Figure 2**).

**Table 3:** Cumulative adversity of township grassroots civil servants and its distribution in other variables.

Cumulative adversity	N(%)		Depression(M ± SD)	Anxiety (M ± SD)
0①	117	9.18	3.82 ± 5.17	1.72 ± 4.13
1②	266	20.86	3.24 ± 5.10	1.84 ± 4.21
2③	258	20.24	5.66 ± 6.38	3.94 ± 5.92
3④	209	16.39	7.35 ± 7.26	5.66 ± 6.83
4⑤	147	11.53	10.97 ± 8.14	8.00 ± 7.71
5⑥	99	7.76	10.06 ± 7.32	7.45 ± 7.90
6⑦	91	7.14	13.20 ± 8.65	9.18 ± 9.20
≥7⑧	88	6.90	17.04 ± 8.56	14.81 ± 10.31
*F*			61.29^***^	50.73^***^
*Post-hoc*			①, ② < ③ < ④ < ⑤, ⑥ <⑦ < ⑧	①, ② < ③ < ④ < ⑤, ⑥, ⑦ < ⑧

***p < 0.001; M ± SD: mean plus or minus the standard deviation; F represents the F-statistic from the analysis of variance (ANOVA); The *Post-hoc* test used was the LSD (Least Significant Difference) method; The > and < symbols indicate a significant difference between groups at a p < 0.05 level. The numbers in parentheses, such as ① and ②, represent the group numbers based on cumulative adversity level. For example, ①, ② <③ means that the score for Group1, 2 are significantly lower than that for Group 3.

**Table 4:** Relationship between cumulative distal and proximal adversity and depression, anxiety.

Depression						Anxiety				
Variable	*R^2^*	*△R^2^*	*F(df)*	β	95% CI	*R^2^*	*△R^2^*	*F(df)*	β	95% CI
Step 1	0.01	0.01	4.11 (5)			0.01	0.01	4.46 (5)		
Age				-0.25	[-0.17, -0.04]				-0.28	[-0.42, -0.14]
Gender				-1.06	[-1.94, -0.18]				-0.62	[-1.45, 0.21]
Household registration				-0.08	[-1.33, 1.15]				0.48	[-0.70, 1.66]
Education				-0.19	[-0.81, 0.43]				-0.40	[-1.01, 0.18]
Step 2	0.24	0.24	69.05 (6)			0.22	0.22	60.10 (6)		
Cumulative adversity (liner term)				1.90^***^	[1.71, 2.09]				1.71^***^	[1.57, 1.89]
Step 3	0.24	0.24	59.14 (7)			0.22	0.21	51.50 (7)		
Cumulative adversity (quadratic term)				0.01	[-0.06, 0.06]				0.01	[-0.05, 0.07]

R²represents the percentage of variance explained by the model; △R^2^ indicates the change in explained variance; F(df) denotes the F-statistic and degrees of freedom for the F-test; β refers to the standardized regression coefficient. 95% CI represents the 95% confidence interval. 95% confidence intervals with predictors were obtained by Bootstrap method. ***p < 0.001.

### 3.5 The mediating role of cumulative proximal adversity

**Figure 3:**
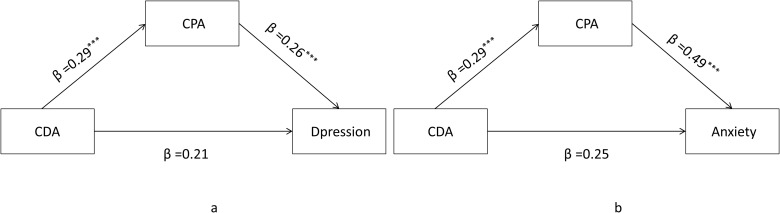
Mediating of cumulative proximal adversity. **(a)** illustrates the mediation model for Depression; **(b)** illustrates the mediation model for Anxiety; CDA-- Cumulative Distal Adversity; CPA -- Cumulative Proximal Adversity, ***p < 0.001.

Correlation analyses confirmed a significant positive relationship between cumulative distal adversity, cumulative proximal adversity, and both mental health outcomes (*Ps* < 0.01; see **Table 5**). As hypothesized, the mediation analysis revealed that the effect of cumulative distal adversity on mental health was fully mediated by cumulative proximal adversity. The indirect effect was significant for both depression (effect = 0.15, 95% CI [0.12, 0.19]) and anxiety (effect = 0.14, 95% CI [0.11, 0.18]), as shown in **Figures 3a, b**.

**Table 5:** Correlation between variables.

Variables	M ± SD	CDA	CPA	Depression	Anxiety
CDA	0.75 ± 1.07	1			
CPA	2.18 ± 1.60	0.29^**^	1		
Depression	7.56 ± 7.94	0.19^**^	0.53^**^	1	
Anxiety	5.45 ± 7.57	0.18^**^	0.48^**^	0.79^**^	1

CDA, Cumulative Distal Adversity; CPA , Cumulative Proximal Adversity; M ± SD: mean plus or minus the standard deviation. **p < 0.01.

## 4 Discussion

This study provides a comprehensive analysis of depression and anxiety among township grassroots civil servants. We examined the prevalence of these mental health outcomes, identified key associated factors, and explored the underlying relationship with cumulative adversity. The findings can help identify individuals at risk and guide the development of targeted prevention efforts for depression and anxiety among township grassroots civil servants

### 4.1 Depression and anxiety among township grassroots civil servants

Our study reveals an alarming mental health situation among township grassroots civil servants, with the overall prevalence of depression and anxiety found to be 36.7% and 29.6%, respectively. This finding of a substantial mental health burden is broadly consistent with previous research in China (10), which has also identified civil servants as a population with high levels of psychological distress. Delving deeper into the pattern of symptoms within our sample, we observed that among the individuals exhibiting symptoms, the moderate severity category was the most frequently reported for both depression and anxiety. However, this phenomenon has not received much attention, especially the township grassroots civil servants of this special group. This may be related to the specific nature of the civil service. Particularly when major events occur, township grassroots civil servants, who are the main implementers of policies, face great time pressure, responsibility pressure, and mental pressure (58). In addition, they are often overloaded with work and have to deal with complex situations (7). These factors are not conducive to the physical and mental health of grassroots civil servants and the stability of grassroots cadres. Therefore, we should pay attention to the current depression and anxiety symptoms among them, and it is necessary to conduct regular mental health check-ups for grassroots civil servants, and provide professional mental intervention and mental counseling for those with mental health problems.

### 4.2 The factors associated with depression and anxiety

Our logistic regression analysis reveals that while many factors are significant, certain proximal adversities demonstrate a disproportionately large impact on mental health. For depression, the strongest predictors were work stress (aOR = 5.02) and economic poverty (aOR = 4.92). For anxiety, the most potent predictors were divorce intention (aOR = 4.92) and parent-child conflict (aOR = 4.53). The powerful effect of these immediate, real-world stressors can be explained by their chronic nature and their direct assault on an individual’s daily coping resources and support systems.

The immense impact of work stress and economic poverty is particularly notable. The work environment of grassroots civil servants often exemplifies the classic “high-demand, low-control” model, a well-established driver of psychological strain and burnout (28). This is not merely a heavy workload but a structural predicament that fosters feelings of helplessness. Simultaneously, economic poverty can trigger a potent sense of relative deprivation; despite their social role, a perceived mismatch between their significant responsibilities and modest income can lead to chronic frustration and a diminished sense of self-worth, which are core pathways to depression (30).

Furthermore, the strong link between family conflict and anxiety highlights the critical role of primary social support. The chronic pressures from their jobs often spill over into the home, creating or exacerbating marital friction and parent-child discord-a clear demonstration of the “stress spillover effect” (29). When the family, which should be a primary buffer against stress, becomes a source of conflict itself, an individual’s core sense of security is eroded. This breakdown of the primary support system leaves them feeling isolated and vulnerable, fostering the hypervigilance and persistent worry that characterize anxiety.

### 4.3 The cumulative adversity on depression and anxiety

A key finding of our study is that the relationship between cumulative adversity and mental health outcomes was linear, suggesting an additive, dose-response pattern. This observation, rather than a curvilinear (accelerating or decelerating) relationship, warrants theoretical consideration as it sheds light on the underlying mechanisms through which adversity impacts this population. One plausible explanation for this linear effect is the coping resource depletion model. This model posits that individuals possess a finite reserve of resources-including psychological resilience, cognitive capacity, social support, and physiological energy-to manage stress (59). Each adverse event, regardless of its specific nature, acts as a drain on this common pool. A linear relationship suggests that each additional stressor depletes these resources by a relatively constant amount, leading to a steady and proportional increase in vulnerability to depression and anxiety (60). In this view, the accumulation of adversity functions like a series of successive blows, each incrementally weakening the individual’s capacity to cope.

This finding is also consistent with the concept of allostatic load, which refers to the cumulative “wear and tear” on the body’s systems from chronic stress (34). A linear effect suggests that each additional adversity contributes a roughly equal measure to this physiological burden, progressively pushing the neurobiological systems that regulate mood and emotion toward a state of dysregulation (36). The absence of an accelerating (exponential) effect might indicate that, for this population, the stressors did not trigger a “kindling” phenomenon, where early adversities sensitize the brain to become hyper-reactive to later events. Similarly, the lack of a decelerating (saturation) effect suggests that even among individuals already facing numerous hardships, each new stressor continued to impose a significant and measurable psychological cost, without reaching a point of diminished impact.

In summary, the observed linear relationship points toward a mechanism of steady, additive risk accumulation. It suggests that for township grassroots civil servants, the sheer quantity of stressors is a powerful, direct driver of mental health problems, progressively overwhelming their coping and physiological systems.

### 4.4 The mediating role of cumulative proximal adversity

Cumulative proximal adversity may be related to symptoms of depression and anxiety by increasing an individual’s stress levels, reducing emotional regulation ability, and affecting cognitive processing styles. Civil servants who are in high-pressure work environments for an extended period may feel fatigued and irritable, which can further lead to symptoms of depression and anxiety (6). Secondly, cumulative distal adversity is associated with a diminished ability to cope with stress, making individuals more vulnerable to the effects of proximal adversity (61, 62). This, in turn, can be related to poorer mental health outcomes (63, 64). For example, experiences of childhood abuse may be associated with difficulties in emotional regulation (65), self-perception (66), and social relationships in adulthood (66), which can be related to a decrease in an individual’s ability to cope with proximal adversity and a higher risk of depression and anxiety symptoms.

Therefore, in predicting and intervening in depression and anxiety symptoms, it is necessary to comprehensively consider the role of both cumulative proximal and distal adversity and adopt multifaceted intervention measures, such as emotional regulation training, cognitive restructuring therapy, and social support enhancement, to improve individuals’ mental health levels and reduce the occurrence of depression and anxiety symptoms.

### 4.5 Theoretical contributions

This study makes several noteworthy contributions to the theoretical understanding of occupational mental health and developmental psychopathology. First, our findings provide strong empirical support for the Developmental Adaptation Model in a new and highly relevant context. By demonstrating the distinct yet interconnected roles of distal (early life) and proximal (current) adversities in shaping the mental health of Chinese grassroots civil servants, we validate the model’s utility beyond Western contexts and highlight its applicability to high-stress occupational groups. Our study specifies how early life vulnerabilities and current stressors interact, confirming the model’s core tenets. Second, this research extends the cumulative risk model by moving beyond confirmation to specification. While many studies have shown that more adversity leads to worse outcomes, a key contribution of our work is the empirical identification of a linear functional form for this relationship. This finding supports an “additive” model of risk, where each additional stressor contributes a relatively equal and significant burden to an individual’s mental health load. This contrasts with curvilinear models and suggests that for this population, there is neither a “kindling” effect (where risk accelerates) nor a “saturation” effect (where risk decelerates). This specificity about the nature of the dose-response relationship is a crucial theoretical refinement. Finally, our study elucidates the mechanistic pathway from early adversity to current psychological distress. The finding that cumulative proximal adversity fully mediates the effect of cumulative distal adversity on both depression and anxiety is a significant theoretical contribution. It suggests that the primary way early life hardships impact adult mental health is not necessarily through a direct, unyielding causal chain, but by increasing an individual’s vulnerability and exposure to contemporary stressors (e.g., work stress, family conflict) (15, 34). This clarifies the “how” in the life course perspective, shifting the theoretical focus toward the interplay between foundational vulnerabilities and immediate environmental pressures.

## 5 Conclusion and implications

This study reveals a high prevalence of depression and anxiety among township grassroots civil servants, underscoring the significant mental health burden faced by this population. Both distal adversities experienced during early life and proximal current stressors demonstrate significant associations with psychological distress. Importantly, proximal adversities fully mediate the relationship between distal adversities and mental health outcomes, highlighting the critical role of present stressors in linking early-life hardships to adult psychological distress.

These findings have important implications for mental health interventions and policy development. Preventive efforts should emphasize early identification and mitigation of childhood trauma to reduce long-term psychological vulnerability. At the same time, addressing current psychosocial stressors-such as work-related stress, economic hardship, and family conflicts-through targeted workplace mental health programs, family support services, and health promotion initiatives is essential. Promoting resilience-building strategies, including cognitive-behavioral therapy, stress management techniques, and social support networks, may alleviate the psychological impact of cumulative adversities.

Overall, a comprehensive, multi-level approach integrating both life-course perspectives and current occupational realities is recommended to effectively improve mental health outcomes among township grassroots civil servants and support the stability and functioning of grassroots governance.

## 6 Limitations and future directions

Several limitations of this study should be acknowledged, which in turn highlight important directions for future research. Firstly, the cross-sectional design precludes causal inference; longitudinal research is required to establish the temporal precedence and directionality of the observed effects. Secondly, the exclusive reliance on self-report measures introduces the potential for recall bias, particularly for distal adversities, and social desirability bias. Future research could enhance validity by employing a multi-method approach that incorporates objective records or informant reports. Thirdly, the non-probability sampling method from a single geographical region may limit the generalizability of the findings, underscoring the need for future studies with more nationally representative samples. Another limitation is our use of the DASS-21, a self-report screening tool, to classify depression and anxiety instead of a formal clinical diagnostic interview. This method is subject to classification bias, including potential false positives and negatives. Therefore, our findings should be interpreted as significant symptomatology, not a formal clinical diagnosis. Future studies should use clinical interviews to confirm these results. Finally, the analytical model did not account for contextual variables; subsequent research could utilize multilevel modeling to explore the influence of organizational and community-level factors on mental health outcomes.

## Data Availability

The raw data supporting the conclusions of this article will be made available by the authors, without undue reservation.
